# Dose-response relationship between volume base dose and tumor local control in definitive radiotherapy for vaginal cancer

**DOI:** 10.1186/s12885-024-12486-1

**Published:** 2024-06-08

**Authors:** Zhiqiang Wang, Xin Guo, Hongfu Zhao

**Affiliations:** Department of Radiation Oncology, China-Japan Union Hospital of Jilin University, No. 126, Xiantai Street, Changchun, 130033 PR China

**Keywords:** Vaginal cancer, Brachytherapy, Dose-response relationship, Tumor control

## Abstract

**Objective:**

This study aimed to establish the dose-response relationship between volume base dose and tumor local control for vaginal cancer, including primary vaginal cancer and recurrent gynecologic malignancies in the vagina.

**Materials and methods:**

We identified studies that reported volume base dose and local control by searching the PubMed, the Web of Science, and the Cochrane Library Database through August 12, 2023. The regression analyses were performed using probit model between volume based dose versus clinical outcomes. Subgroup analyses were performed according to stratification: publication year, country, inclusion time of patients, patients with prior radiotherapy, age, primaries or recurrent, tumor size, concurrent chemoradiotherapy proportion, dose rate, image modality for planning, and interstitial proportion.

**Results:**

A total of 879 patients with vaginal cancer were identified from 18 studies. Among them, 293 cases were primary vaginal cancer, 573 cases were recurrent cancer in the vagina, and 13 cases were unknown. The probit model showed a significant relationship between the HR-CTV (or CTV) D90 versus the 2-year and 3-year local control, *P* values were 0.013 and 0.014, respectively. The D90 corresponding to probabilities of 90% 2-year local control were 79.0 Gy_EQD2,10_ (95% CI: 75.3–96.6 Gy_EQD2,10_).

**Conclusions:**

A significant dependence of 2-year or 3-year local control on HR-CTV (or CTV) D90 was found. Our research findings encourage further validation of the dose-response relationship of radical radiotherapy for vaginal cancer through protocol based multicenter clinical trials.

**Supplementary Information:**

The online version contains supplementary material available at 10.1186/s12885-024-12486-1.

## Introduction

Primary vaginal cancer is a rare cancer, with an estimated 17,600 new cases and 8,062 deaths worldwide in 2020 [1]. Due to the relative rarity of primary vaginal cancer, prospective evaluation of its management is difficult. Fortunately, retrospective studies have demonstrated successful treatment of primary vaginal cancer with definitive radiotherapy, including external beam radiotherapy (EBRT) followed by image-guided brachytherapy (IGBT) [2–5]. For patients with early gynecological malignancies, such as cervical cancer and endometrial cancer, etc., radical hysterectomy achieved excellent cure rates. Radiotherapy is an important treatment option for medically inoperable endometrial cancer and locally advanced cervical cancer [6, 7]. However, after radical hysterectomy or radiotherapy, 10–20% of patients experience recurrence, with the majority still limited to the pelvis [8, 9]. The vagina is an important site of recurrence. Vaginal recurrences from gynecological malignancies pose clinical challenges. Organ-preserving approaches with EBRT and IGBT play an important roles in the treatment of vaginal recurrence from endometrial cancer and cervical cancer [10–13]. It has been shown that brachytherapy (BT) can improve survival and is an important component of definitive radiotherapy in vaginal cancer [14, 15].

Since 2005, GEC-ESTRO has released recommendations for three-dimensional brachytherapy of cervical cancer, which not only had a profound impact on the brachytherapy of cervical cancer, but also its methods have been borrowed by vaginal cancers [16–19]. The consensuses on target volume delineation for primary vaginal cancer [20] and recurrent endometrial and cervical tumors in the vagina [10] have only recently been reached. The high risk clinical target volume (HR-CTV) includes the residual gross tumor volume (GTV) and areas on imaging and/or clinical examination that are concerning for harboring macroscopic pathologic disease. It is admirable that before reaching these consensuses, many medical institutions had already started image-guided vaginal cancer brachytherapy and reported volume related doses. Although some researchers did not use names like HR-CTV and only used clinical target volume (CTV), the two names point to similar definitions. After analyzing the data of 91 cases of primary squamous cell carcinoma (SCCA) of the vagina treated with definitive radiotherapy, Hiniker et al. concluded that the optimal dose for definitive treatment of SCCA of the vagina lies between 70 and 80 Gy [21]. Nevertheless, the optimal volume based dose and fractionation plans have not been well-known up till now.

For cervical cancer, we have previously analyzed the dose-response relationships in image-guided BT [22], three-dimensional intracavitary combined with interstitial BT [23], and four-dimensional adaptive BT [24], and obtained results consistent with current consensus of dose constraints [25]. The purpose of this study is to identify articles that reported volume base dose and local control in definitive radiotherapy for vaginal cancer through systematic literature screening, and to conduct probit model analysis in an attempt to find the optimal dose for definitive radiotherapy for vaginal cancer.

## Materials and methods

### Data sources and search strategy

We performed a comprehensive literature search using the PubMed, the Web of Science, and the Cochrane Library Database to identify full articles reported the volume base dose and local control of brachytherapy in patients with vaginal cancer, including primary vaginal cancer and vaginal recurrence from gynecologic malignancies. The last search of this systematic search was performed on August 12, 2023. We searched MeSH terms “Vaginal Neoplasms” and “brachytherapy” or their all Entry terms in the title or abstract, and the search was restricted for English-language, see Supplemental Table 1. References from system reviews, guidelines, or recommendations are also included in the literature screening and eligibility process. We contacted the corresponding authors when full-text articles were not available.

### Inclusion criteria were as follows

1. Original articles reported EBRT with or without concurrent chemotherapy and IGBT for patients with vaginal cancer, including primary vaginal cancer and vaginal recurrence from gynecologic malignancies;
2. Articles reported sufficient data for probit regression analysis, including equivalent dose in 2 Gy per fraction (EQD2), using the linear quadratic model, with α/β = 10 Gy, for minimum doses delivered to 90% (D90) of HR-CTV (or CTV) and local control rate;
3. There was no limitation on nationality, race, age, stage.

### Exclusion criteria were as follows

1. Conference abstracts without full-text;
2. Review articles, articles about recommendations, consensuses or guidelines;
3. Irrelevant literature or literature focused on technique, dosimetry, side effect, quality of life etc.;
4. Insufficient data;

### Data extraction

Two authors screened the titles, abstracts and full-texts independently. Data in all enrolled studies were extracted according to the following procedures: (1) study information: first author, publication year and country; (2) patients characteristics: inclusion time of patients, sample size, patients number with prior radiotherapy and median age; (3) tumors characteristics: primary or recurrent, median tumor size prior to BT; (4) treatment characteristics: technique of EBRT, concurrent chemotherapy proportion, dose rate, fractionation, applicator, image modality for planning, interstitial proportion; (5) dose-volume histogram (DVH) parameters: EBRT dose, HR-CTV or CTV D90; (6) clinical outcomes: median follow-up, local control rate, disease-free survival (DFS) rate, and overall survival (OS) rate. Data were independently extracted by two authors from all eligible studies following the inclusion criteria and the exclusion criteria. Discrepancies were resolved by consultation with a third author.

### Data analysis

The regression analyses between volume based dose and clinical outcomes were performed using probit model by XLSTAT 2016 (Addinsoft, Paris, France). Mean or median value was selected as the quantitative dose. The number of patients reported was selected as an Observation Weight to consider the influence of sample size. Statistical significance set at the *P* < 0.05 level.

Subgroup analyses were performed according to stratification: publication year, country, inclusion time of patients, patients with prior radiotherapy, age, primaries or recurrent, tumor size, concurrent chemoradiotherapy proportion, dose rate, image modality, and interstitial proportion.

## Results

### Description of the included studies

After comprehensive searching, no published regression analyses on dose-response relationship between dose and local control was identified. We used the systematic literature search strategy, and 1,232 potentially relevant studies were identified.

Prior to screening the title and abstract and reviewing the full-text, duplications were checked, 18 studies were enrolled in the dose-response analysis, see Supplemental Fig. 1.

### Probit analyses

One of the 18 included studies in our analysis was a multicenter study from Denmark, France, the Netherland, and Vienna [5]. Besides, the others studies were from 7 countries, with most publications being from the United States of American (*n* = 10) [11, 26–34], followed by the India (*n* = 2) [12, 13], Austria [35], Canada [3], Denmark [36], France [4], and Japan [37] (one each). The main characteristics of the 18 included studies are presented in Tables 1 and 2.

**Table 1:** Characteristics of the included studies: study information, patient’s characteristics, tumors characteristics, and external beam radiotherapy

First Author & Publication Year	Country	Inclusion Time of Patients	Sample Size	Patients *N*. with Prior RT	Median Age (y)	Primary or recurrent	Median Tumor Size Prior to BT	Technique of EBRT	EBRT Dose (Gy)
Beriwal 2012 [26]	USA	2000–2010	30	5	66 (44–89)	17 primaries, 13 recurrent	39.3 ± 25.7 cc; 3.3 (1.3-8.0) cm	NR	45 (24-50.4) Gy in 12–28 f
Dimopoulos 2012 [35]	Austria	1999–2006	13	0	59 (33–80)	NR	10.2 (2.0-43.2) cc	4-field 3D-CRT, 25 MV LA	45–50.4 Gy, 1.8–2.0 Gy/f, 10–15 Gy boost to node
Lee 2013 [27]	USA	2003–2011	(a) 31 (b) 13	(a) 0 (b) 13	66 (49–88)	(a) 31 recurrent (b) 13 recurrent	2.1 (0–7) cm	NR	45 Gy (40-50.4), 18 Gy boost to node
Fokdal 2014 [36]	Denmark	2006–2013	43	0	71 (38–83)	43 recurrent	18 (0–91) cc	28 3D-CRT, 15 IMRT	45–50 Gy in 25–30 f, SIB 60 Gy to node
Vargo 2014 [28]	USA	2004–2013	41	0	66 (33–81)	41 recurrent	2.6 (0-7.5) cm	36 IMRT	45–50.4 Gy in 25–28 f, SIB 55 Gy to node
Vargo 2015 [29]	USA	2011–2014	41	0	67 (35–87)	10 primaries, 31 recurrent	24.2 IQR 12.6 cc; 2.0 (0.5–5.7) cm	41 IMRT	44–50.4 Gy, 1.8–2 Gy/f, SIB 55 Gy to node
Chapman 2017 [30]	USA	2000–2010	30	0	73 (57–94)	30 recurrent	28.9 (17.6–76.6) cc for13 available plans	NR	1.8 Gy/f, 25 (25–28) f
Kamran 2017 [11]	USA	(a) 2005–2016 (b) 2011–2016	(a) 18 (b) 48	(a) 9 (b) 15	(a) 68.0 (41.2–81.2) (b) 63.6 (34.7–83.7)	(a) 18 recurrent (b) 48 recurrent	(a) 39% >4 cm (b) 15% >4 cm	(a) 7 IMRT, 7 3D-CRT, 4 others (b) 11 IMRT, 24 3D-CRT, 13 others	(a) 44.3 (30.1–50.0) (b) 44.3 (20.6–46.0)
Gebhardt 2018 [31]	USA	2011–2016	60	0	66 (35–87)	16 primaries, 44 recurrent	2.0 (0.5–5.8) cm, 24.4 IQR 14.1 cc	57 IMRT	44–50.4 Gy, 1.8–2 Gy/f, SIB 55 Gy to node
Huertas 2018 [4]	France	2004–2017	27	0	56 (23–75)	27 primaries	16.1 (0.6–71.5) cc	7 IMRT, 20 3D-CRT	45 Gy in 25f, 60 Gy to node
Ling 2019 [32]	USA	NR	22	22	71 (79–90)	22 recurrent	23.2 (IQR 13.0–30.6) cc	11 EBRT	45.0 (30.6–50.4) Gy in 25 f, 60 Gy boost or 55 Gy SIB to node
Chopra 2020 [13]	India	2011–2016	50	0	47 (35–65)	50 recurrent	38 (12–85) cc	3D-CRT or IMRT	50 Gy/25f, 54–55 Gy SIB to node
Patel 2020 [33]	USA	2014–2020	13	3	58 (30–83)	3 primaries, 10 recurrent	0.71 (0-6.16) cm	NR	44.4 Gy in 24f, SIB 59.4 (56.3–62.5) Gy to node
Alban 2021 [34]	USA	2004–2017	62	0	64.6 (35.9–85.1)	62 recurrent	2.5 (0.3-8) cm ^*^	3D-CRT or IMRT	45 (44-50.4) Gy, 63 (54–70) Gy boost to node
Goodman 2021 [3]	Canada	2002–2017	67	0	68 (IQR 56–75)	67 primaries	4.1 ± 1.5 cm for 55 patients	3D-CRT or VMAT	45 Gy in 25f
Murofushi 2021 [37]	Japan	2010–2015	22	0	63 (33–78)	22 recurrent	17 (0–45) mm	17 3D-CRT	30.0–50.0 Gy in 15–25 f + 30.0–50.0 Gy with MB
Westerveld 2021 [5]	Multicenter ^**^	2014–2017	148	0	63 (IQR54-73)	148 primaries	17.6 (IQR 6.8–32.1) cc	90 3D-CRT; 55 IMRT/VMAT	45.0–50.4 Gy in 1.7–2.0 Gy/f, 60–64 Gy to node
Engineer 2022 [12]	India	2008–2014	90	0	50	90 recurrent	46 patients >4 cm	90 Tomotherapy	50 (46–50) Gy/25 (23–25) f, 55–60 Gy SIB to node

^*^ Tumor size at EBRT

^**^ The Netherland, Vienna, France, Denmark

N. = number; RT = Radiotherapy; y = year; EBRT = external beam radiotherapy; USA = United States of American; NR = not reported; 3D-CRT = three-dimensional conformal radiotherapy; LA = linear accelerator; IMRT = intensity modulated radiotherapy; IQR = interquartile range; VMAT = volumetric modulated arc therapy

**Table 2:** Characteristics of the included studies: concurrent chemoradiotherapy, brachytherapy characteristics, volume based dose, and clinical outcomes

First Author & Publication Year	Concurrent Chemo-RT (%)	Dose Rate	Fractionation	Applicator	Image Modality	IS Proportion	Median D90 (Gy_EQD2,10_)	Median (Range) Follow-up (m)	LC rate	DFS rate	OS rate
Beriwal 2012 [26]	53.3%	HDR	4.25 (3.75-5.0) Gy/f, 5f	SN template-based IS	CT	100.0%	74.3 (36.3–81.1)	16.7 (0.9–52.9)	1-y: 84.4%; 2-y: 78.8%	NR	1-y: 82.1%; 2-y: 70.2%
Dimopoulos 2012 [35]	84.6%	3 HDR; 8 PDR; 2 HDR + PDR	HDR: 5–8 Gy/f, 2–6 f; PDR: 32 (20–42) Gy	1 IV; 12 IV and IS	MRI	92.3%	86 ± 13	43 (19–87)	3-y: 92%	NR	3-y: 85%
Lee 2013 [27]	20.4%	(a) 26 HDR; 5 LDR (b) 12 HDR; 1 LDR	HDR cylinder: 4.8 Gy/f 3–9 f; LDR IS: 0.53 Gy/h 0.3–0.65; HDR IS: 4 Gy/f 2.5-6 f	(a) 8 cylinder; 23 SN based IS (b) 1 cylinder; 12 SN based IS	10 MRI; 34 CT	(a) 74.2% (b) 92.3%	(a) 74.8 (52.0-100.0) (b) 59.8 (30.9–85.7)	24 (2–88)	(a) 2-y: 96% (b) 2-y: 61%	(a) 2-y: 72% (b) 2-y: 26%	(a) 2-y: 80% (b) 2-y: 55%
Fokdal 2014 [36]	12.0%	PDR	10–17.5 Gy in 10–20 hourly pulses	19 MCVC; 24 MUPIT based IS	19 CT, 24 MRI	55.8%	82.0 (77–88)	30 (6–88)	2-y: 92%	2-y: 59%	2-y: 78%
Vargo 2014 [28]	19.0%	HDR	20–25 Gy in 4–5 f	9 SCVC, 21 MCVC, 11 template based IS	CT and/or MRI 12 CT + MRI	27.0%	76 (61.3–83.2)	18 (3–78)	3-y: 95%	3-y: 68%	3-y: 67%
Vargo 2015 [29]	NR	HDR	25 (20-27.5) Gy in 5 f	MCVC	18 CT, 23 MRI	0.0%	77.1, IQR 3.4	16 (3–35)	2-y: 93%	2-y: 78%	2-y: 88%
Chapman 2017 [30]	10.0%	HDR	6 (5–10) Gy/f, 3 (2–3) f	27 cylinder with IS, 3 cylinder	CT	90.0%	70.8	76.4 (10.8-149.2)	5-y: 87%	2-y: 79.0%; 5-y: 75.0%	2-y: 80.0%; 5-y: 77.0%
Kamran 2017 [11]	(a) 33.0% (b) 42.0%	HDR	(a) 5.0 (3.3–6.7) Gy/f, 5 (4–9) f (b) 4.5 (2.3-8.0) Gy/f, 5 (3–9) f	IS catheters	(a) MRI (b) CT	100.0%	(a) 75.7 (58.6-108.7) (b) 75.2 (37-104.2)	(a) 35 (4–56) (b) 30 (3-103)	(a) 2-y: 100%; 3-y: 100% (b) 2-y: 78%; 3-y: 78%	(a) 3-y: 69% (b) 3-y: 55%	(a) 3-y: 63% (b) 2-y: 75%
Gebhardt 2018 [31]	32.0%	HDR	4.0–5.5 Gy/f, 4–5 f	MCVC	41 MRI, 19 CT	0.0%	77.2 IQR 2.8	45 (11–78)	2-y: 92.6%; 4-y: 92.6%	2-y: 75.0; 4-y: 64%	4-y: 67.2%
Huertas 2018 [4]	85.0%	PDR	1 (1–2) f, 30 to 60 pulses	Vaginal mold ± IS	22 MRI	59.0%	73.1 (52-112.5)	40.1 (5.4–86.8)	2-y: 82%; 3-y: 82%	2-y: 70%; 3-y: 65%	2-y: 86%; 3-y: 86%
Ling 2019 [32]	13.6%	HDR	28.75 Gy (IQR: 24.8–30) in 4–7 f	1 SCVC; 8 IS; 2 MCVC with free hand needles; 11 MCVC;	5 CT; 17 MRI	36.4%	64.5 (IQR49.5-75.8)	27.6 (IQR 7.5–50)	2-y: 65.8%; 3-y: 65.8%	2-y: 40.8%; 3-y: 40.8%	2-y: 82.5%; 3-y: 68.1%
Chopra 2020 [13]	88.0%	HDR	20 (12–20) Gy/ 2–5 f	10 SCVC; 2 MCVC; 35 MUPIT based IS	MRI + PET + CT	70.0%	71.0 (50–74)	60 (5–93)	2-y: 91.0%; 3-y: 91.0%; 5-y: 84.0%; 7-y: 84.0%	2-y: 84.6%; 3-y: 82.0%; 5-y: 73.0%; 7-y: 70.0%	2-y: 85.6%; 3-y: 83.0%; 5-y: 74.5%; 7-y: 70.6%
Patel 2020 [33]	15.4%	HDR	25.5 Gy in 3–5 f	Modified MIAMI MCVC	CT + pre-implant MRI	100.0%	81.2 (52.9–95.5)	15.1 (1–49)	3-y: 92.0%	NR	3-y: 92.0%
Alban 2021 [34]	17.0%	59 HDR; 3 LDR	HDR: 25 (16–35) Gy; LDR:32.3 (30–35) Gy	cylinder or SN based IS	15 MRI; 47 CT	58.1%	75.3 (61.5–98.1)	39 (3-167)	2-y: 91.0%; 3-y: 86.0%; 5-y: 82.0%	2-y: 76.0%; 3-y: 69.0%; 5-y: 55.0%	2-y: 92.0%; 3-y: 80.0%; 5-y: 60.0%
Goodman 2021 [3]	70.2%	HDR	6.5 (6–7) Gy/f, 3 (3–4) f	MCVC with template-based IS	CT and or MRI	100.0%	74.0	32.4 (95CI 24–72)	2-y: 87.5%; 3-y: 84.5%	2-y: 73.5%; 3-y: 66.4%	2-y: 86.5%; 3-y: 81.7%
Murofushi 2021 [37]	31.8%	HDR	18–35 Gy in 3–5 f	10 cylinder or ovoids; 12 MUPIT based IS	CT	54.5%	69.2 (62.6–72.8) Gy	58.7 (9.6–93.1)	2-y: 91%; 5-y: 95.5%	5-y: 77.3%	2-y: 84.8%; 5-y: 84.8%
Westerveld 2021 [5]	64.0%	115 PDR; 33 HDR	HDR: 6–7 Gy/f 3f	MCVC with tandem; or plus needles	42 CT; 77 MRI; 29 MRI + CT	55.0%	80 (IQR 73.0–85.2) Gy	29 (IQR 25–57)	2-y: 86%; 5-y: 83%	2-y: 73%; 5-y: 66%	2-y: 79%; 5-y: 68%
Engineer 2022 [12]	100.0%	HDR	20 (14–24) Gy in median 5f, twice a day	MUPIT based IS	CT	100.0%	72 (46–82)	74 (4-123)	2-y: 90.0%; 7-y: 87.6%	2-y: 83.2%; 7-y: 68.3%	2-y: 82.1%; 7-y: 68.3%

IS = interstitial; EBRT = external beam radiotherapy; m = month; LC = local control; DFS = disease free survival; OS = overall survival; HDR = high dose rate; f = fraction; SN = Syed-Neblett; CT = computed tomogram; y = year; NR = not reported; PDR = pulsed dose rate; IV = intravaginal; MRI = magnetic resonance image; LDR = low dose rate; MCVC = multichannel vaginal cylinder; MUPIT = Martinez universal perineal interstitial template; SIB = simultaneous integrated boost; SCVC = single channel vaginal cylinder; IQR = interquartile range; MB = midline block

**Fig. 1:**
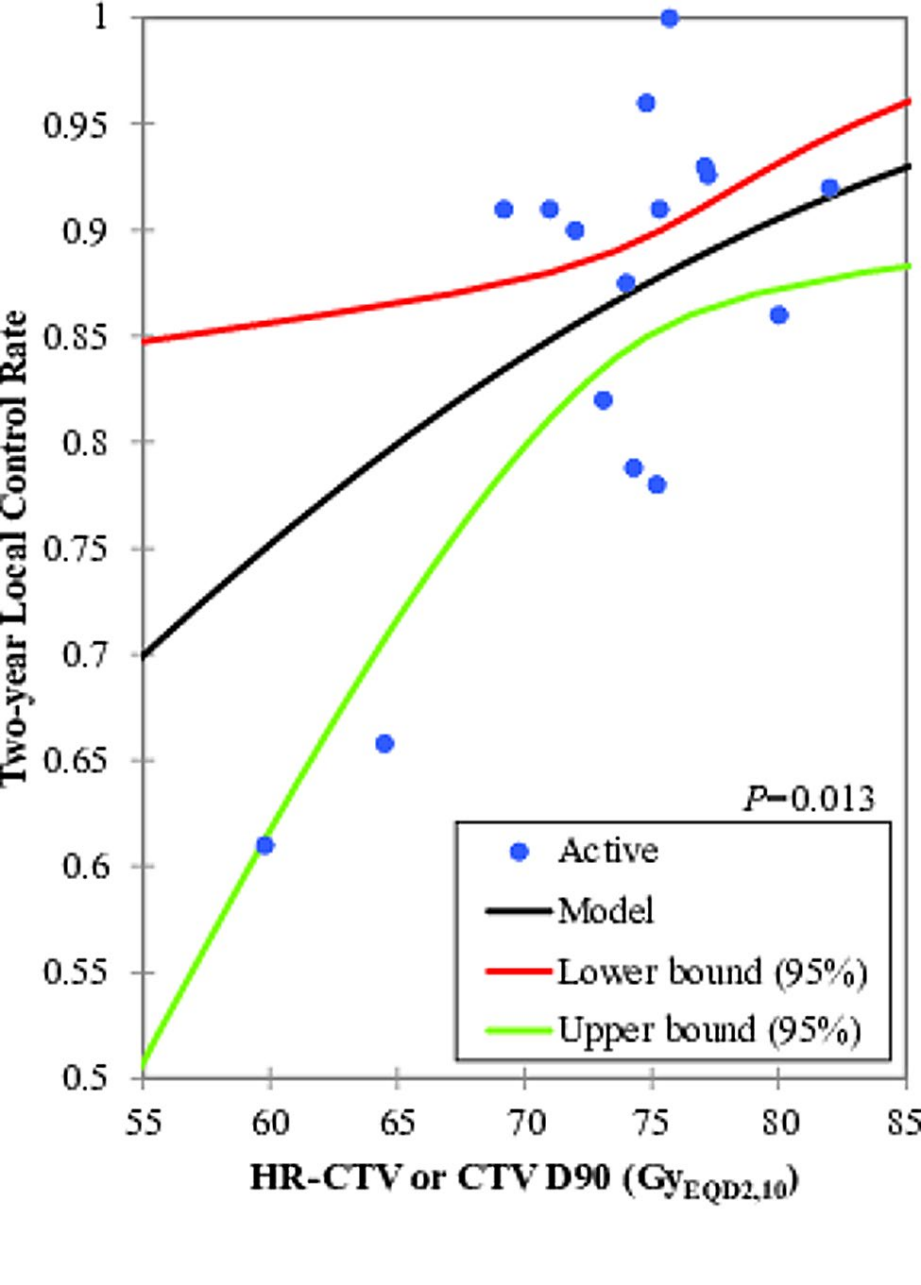
The probit model for the relationship between HR-CTV (or CTV) D90 and two-year local control. The blue dots represent the values of D90 and the two-year local control for each study

**Fig. 2:**
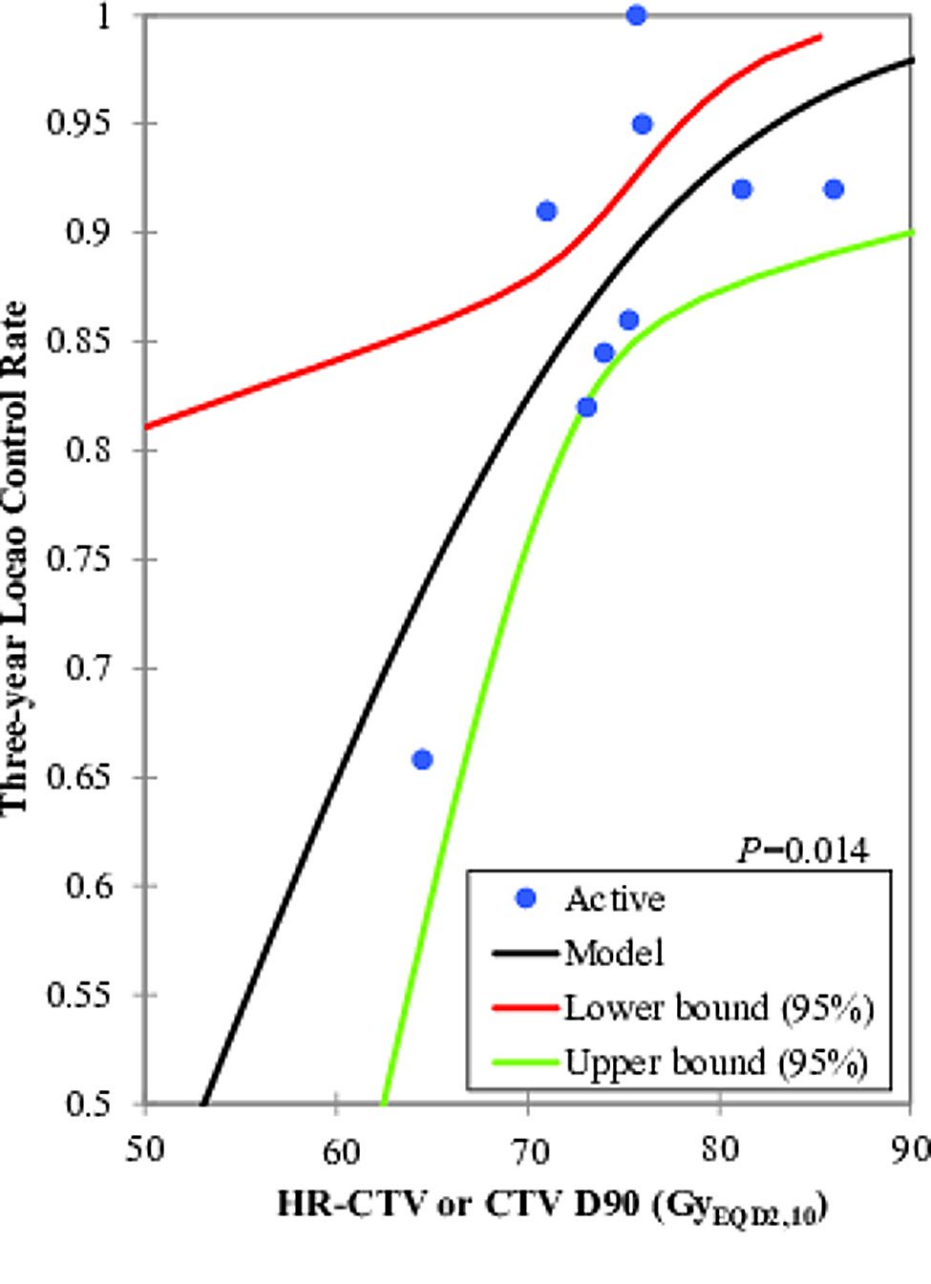
The probit model for the relationship between HR-CTV (or CTV) D90 and three-year local control. The blue dots represent the values of D90 and the three-year local control for each study

The mean or median of HR-CTV D90 or CTV D90 were reported from 59.8 to 86.0 Gy_EQD2,10_, and actuarial or crude 2-year, 3-year, and 5-year local control rates were reported to be 61.0–100.0%, 65.8–100.0%, and 82.0–95.5%, respectively. The probit model showed significant relationships between the HR-CTV (or CTV) D90 versus the 2-year and 3-year local control, *P* value were 0.013 and 0.014, respectively (Figs. 1 and 2). According to this model, the D90 corresponding to probabilities of 80%, 90%, and 95% local control were 65.1 Gy_EQD2,10_ (95% confidence interval (CI): 28.9–70.2 Gy_EQD2,10_), 79.0 Gy_EQD2,10_ (95% CI: 75.3–96.6 Gy_EQD2,10_) and 90.5 Gy_EQD2,10_ (95% CI: 83.0–149.1 Gy_EQD2,10_), respectively. The prescribed dose to HR-CTV (CTV) D90 of 75 and 80 Gy_EQD2,10_ would in theory warrant a 2-year local control rate of 87.1% (95% CI: 91.8% − 90.0%) and 90.4%, (95% CI: 83.7 − 93.0%), respectively. There was no significant dose response relationship between HR-CTV or CTV D90 versus DFS and OS probability, *P* values were 0.167 and 0.788, respectively (Table 3). The results of subgroup analysis based on stratification are shown in Table 4.

**Table 3:** The probit model results between volume base dose and clinical outcome

Clinical outcome	Studies	Patients	ED90 (95% CI) (Gy_EQD2,10_)	SE	Chi-square	*P*
Local control						
2-year	14	772	79.0 (75.3, 96.6)	0.013	6.190	**0.013**
3-year	9	313	76.3 (73.1, 90.0)	0.023	5.982	**0.014**
5-year	5	312	63.0 (-, -)	0.021	1.024	0.312
Disease-free survival						
2-year	12	684	118.6 (-, -)	0.011	1.914	0.167
3-year	7	394	163.3 (-, -)	0.015	0.385	0.535
5-year	5	312	37.0 (-, -)	0.018	1.591	0.208
Overall survival						
2-year	14	724	193.8 (-, -)	0.012	0.073	0.788
3-year	9	313	107.0 (-, -)	0.019	0.591	0.444
5-year	5	312	48.3 (-, -)	0.018	2.371	0.124

ED90 = estimated dose at 90%, CI = confidence interval, SE = standard error

*P*-value in bold represents that the probit model has statistical significance

**Table 4:** The probit model for subgroup between HR-CTV or CTV D90 and 2-year local control

Parameter	Studies	Patients	ED90 (95% CI) (Gy_EQD2,10_)	SE	Chi-square	*p*
Publication year						
2012–2017	7	224	78.1 (74.2, 88.3)	0.020	7.698	**0.006**
2018–2022	9	548	82.1 (-, -)	0.016	0.943	0.331
Country						
USA	9	325	76.2 (73.7, 80.5)	0.018	15.265	**< 0.0001**
Others	7	447	65.6 (-, -)	0.018	0.275	0.600
Inclusion time of patients						
Before 2015	7	270	74.7 (69.4, 87.9)	0.020	5.574	**0.018**
After 2010	6	369	86.5 (-, -)	0.024	0.394	0.530
Patients with prior radiotherapy						
≤ 10%	11	641	72.9 (-, -)	0.018	0.207	0.649
> 10%	5	131	82.3 (75.8, 156.9)	0.021	5.123	**0.024**
Median age						
≤ 65	7	447	68.4 (-, -)	0.021	1.029	0.310
> 65	9	325	75.8 (73.0, 79.9)	0.016	16.288	**< 0.0001**
Primary vaginal cancer						
≤ 20%	11	466	75.2 (72.3, 81.7)	0.017	9.445	**0.002**
> 20%	5	306	84.9 (-, -)	0.037	0.310	0.578
Tumor size ^*^						
≤ 30 mm	6	229	73.1 (67.7, 77.9)	0.022	7.963	**0.005**
> 30 mm	10	543	84.6 (-, -)	0.015	1.803	0.179
CCRT proportion						
≤ 50%	9	319	76.1 (73.0, 81.5)	0.016	12.922	**< 0.0001**
> 50%	7	453	65.8 (-, -)	0.022	0.392	0.531
Dose rate						
HDR > 80%	13	554	75.0 (72.7, 79.5)	0.017	11.325	**0.001**
Image modality for planning						
CT > 50%	7	296	77.1 (-, -)	0.024	2.832	0.096
MRI > 50%	7	359	80.1 (76.0, 97.1)	0.018	5.557	**0.018**
Interstitial proportion						
> 50%	13	649	83.2 (-, -)	0.014	0.323	0.250

^*^ The tumor volume reported were converted to tumor diameter using the spherical volume formula

ED90 = estimated dose at 90%, CI = confidence interval, SE = standard error, USA = the United States of American, CCRT = concurrent chemoradiotherapy, HDR = high dose rate, CT = computed tomography, MRI = magnetic resonance imaging. *P*-value in bold represents that the probit model has statistical significance

## Discussion

Definitive radiotherapy is one of important treatment options for vaginal cancer, whether primary or recurrent, as it can preserve organ function and improve quality of life. However, so far, there is no consensus on the optimal prescription dose of definitive radiotherapy for vaginal cancer. Our study fills the gap in this regard. The studies of the dose toxicity relationship of the vagina as a normal tissue have provided dose constraints for clinical practice [38–40]. Combined with the results of this study, radiation oncologists can seek an optimal window for achieving high local tumor control while maintaining low side effects for OARs.

Our study demonstrated that two-year tumor control probability of > 90% can be expected at doses > 79.0 Gy_EQD2,10_. This result is consistent with previous study by Hiniker et al [21]. After retrospectively analyzed the data of 91 patients with primary squamous cell carcinoma (SCCA), Hiniker et al. concluded that the optimal dose for definitive treatment of SCCA of the vagina lies between 70 and 80 Gy. In their study, a total radiation dose of > 70 Gy was associated with improved local disease control and a trend towards improved OS. It is worth noting that the dose used in their study is the prescription dose, which is the dose at depth of 5 mm or at vaginal mucosa, while the dose used in our study is volume based dose, which is D90. However, there was no significant dose-response relationship observed in our probit model analysis of the subgroup of primary vaginal cancer. This may come from two reasons. On the one hand, there was only 5 studies with a proportion of patients with primary vaginal cancer exceeding 20% in the included studies, involving 306 patients. On the other hand, the concentration of data is a reason why probit model analysis is not easy to achieve.

In vaginal cancer, there are several guidelines that provide prescription doses that are consistent with the optimized dose provided by the probit model in our study. The American Brachytherapy Society consensus guidelines for interstitial brachytherapy for vaginal cancer in 2012 stated that for disease involving the distal vagina in close proximity to the vulva or rectovaginal septum, consideration should be given to a total dose of 70–75 Gy; patients who have had poor response to EBRT or have large residual disease may benefit from higher total dose of 80–85 Gy [41]. The doses here are still prescription dose, not volume based dose. Moreover, the setting of prescription dose is mainly based on the consideration of dose tolerance to OARs, rather than considering the probability of tumor control. In the ESTRO/ESGO/SIOPe guidelines for the management of patients with vaginal cancer, the planning aim for the total dose of EBRT + BT was equal to or greater than 75 to 85 Gy to HR-CTV D90 [42]. Our probit model aligns well with this planning aim. According to our model, the HR-CTV D90 of 75 Gy to 85 Gy was expected to achieve a 2-year local control of 87–93%. The French recommendation for primary vaginal cancer stated that the dose of HR-CTV D90 should be at least 70–75 Gy, and the dose should be escalated to 80 Gy on a case-by-case basis, particularly for tumors in the upper third of the vagina [43].

In our study, there was no significant dose-response correlation between volume based dose and DFS and OS, whether it was 2-year, 3-year, or 5-year. This may be due to the heterogeneity of enrolled patients. After all, radiotherapy is a local physical therapy method, so it has the strongest correlation with local control. In the study of dose-response relationship for cervical cancer, there were also significant dose-response relationships between volume based dose and OS and cancer specific survival (CSS). Zhang et al. conducted a dose-response analysis on the data of 110 patients with locally advanced cervical cancer treated with radical concurrent chemo-radiotherapy combined with intracavitary and interstitial brachytherapy, and still obtained the dose-response correlation between HR-CTV based dose and OS and CSS [44]. Similarly Ke et al. obtained the significant dose-response relationship between GTV based dose and OS and CSS [45].

Our study included 573 patients with recurrent vaginal cancer, of which 67 had a history of radiotherapy. This made us have to think about a question, which is whether patients with a history of radiotherapy can accept the prescribed dose obtained from the probit model analysis? In terms of tumor control, newly grown tumors after previous radiotherapy have not been exposed to previous radiation, so the recurrent vaginal cancer patients with a history of radiotherapy should accept the optimized prescribed dose. However, the surrounding OARs were severely hit by both two courses of radiotherapy. When evaluating the risk of side effects, a comprehensive consideration should be given to the cumulative dose from two courses of radiotherapy and the interval time between two courses of radiotherapy to avoid serious side effects. Zolciak-Siwinska et al. [46] found that a cumulative EQD2 of approximately 100 Gy was safely delivered to D2cc of the bladder and the rectum. Ling et al.‘s study once again confirmed that re-irradiation with 3D conformal brachytherapy for vaginal recurrence was feasible and safe as long as cumulative dose to surrounding normal organs was limited [32] 0. A recent multicenter survey from Japan showed that a higher cumulative EQD2 was significantly associated with severe complications [47]. In term of interval time, Paradis et al. [48] proposed a systematic approach to the re-irradiation special medical physics consult process, which provided a previous dose discount related to interval time. Taking the bladder and rectum as an example, interval time of < 3 months, 3 months − 6 months, 6 months − 1 year, and 1 year − 3 years correspond to previous dose discount 0%, 10%, 25%, and 50%, respectively.

In radiotherapy, dose-response relationships were objective and widely recognized. Before the establishment of the dose-response curve, the optimal prescription dose for the target volumes was unclear, and it was more or less influenced by the dose constraints of the OARs. Our study derived significant dose-response relationships between volume based dose and local control based on published research results. The establishment of this dose-response relationship clears the fog for future clinical practice, striving to achieve the optimal dose recommended by the dose-response relationship while maintaining a controllable risk of OARs. Although our results are preliminary, to our knowledge, it is the first dose-response relationship study for radical radiotherapy of vaginal cancer.

Like many other studies, this study has some limitations. Firstly, there were certain differences in the delineation and naming of target volumes for included studies, which was due to the consensuses had only been reached in recent years regarding the delineation of target volumes for vaginal cancer [10, 20]. Secondly, the total number of patients included in the probit analysis was not high due to the relative rarity of vaginal cancer, and they were all observational studies. Finally, the heterogeneity of the data remains an important limitation of this study, as previously stated [22–24].

In conclusion, a significant dependence of 2-year or 3-year local control on HR-CTV (or CTV) D90 was found. Two-year tumor control probability of > 90% can be expected at doses > 79.0 Gy_EQD2,10_ based on meta-regression analysis. Our research findings encourage further validation of the dose-response relationship of radical radiotherapy for vaginal cancer through protocol based multicenter clinical trials.

### Electronic supplementary material

Below is the link to the electronic supplementary material.

Supplementary Material 1

Supplementary Material 2

## Data Availability

All data, models, or code generated or used during the study are available from the corresponding author by request.
