# Supplementary figures and images for: Dose-response relationship between volume base dose and tumor local control in definitive radiotherapy for vaginal cancer
Source: BMC Cancer. 2024 Jun 8;24:707. doi: 10.1186/s12885-024-12486-1 (PMC11162573; doi:10.1186/s12885-024-12486-1)

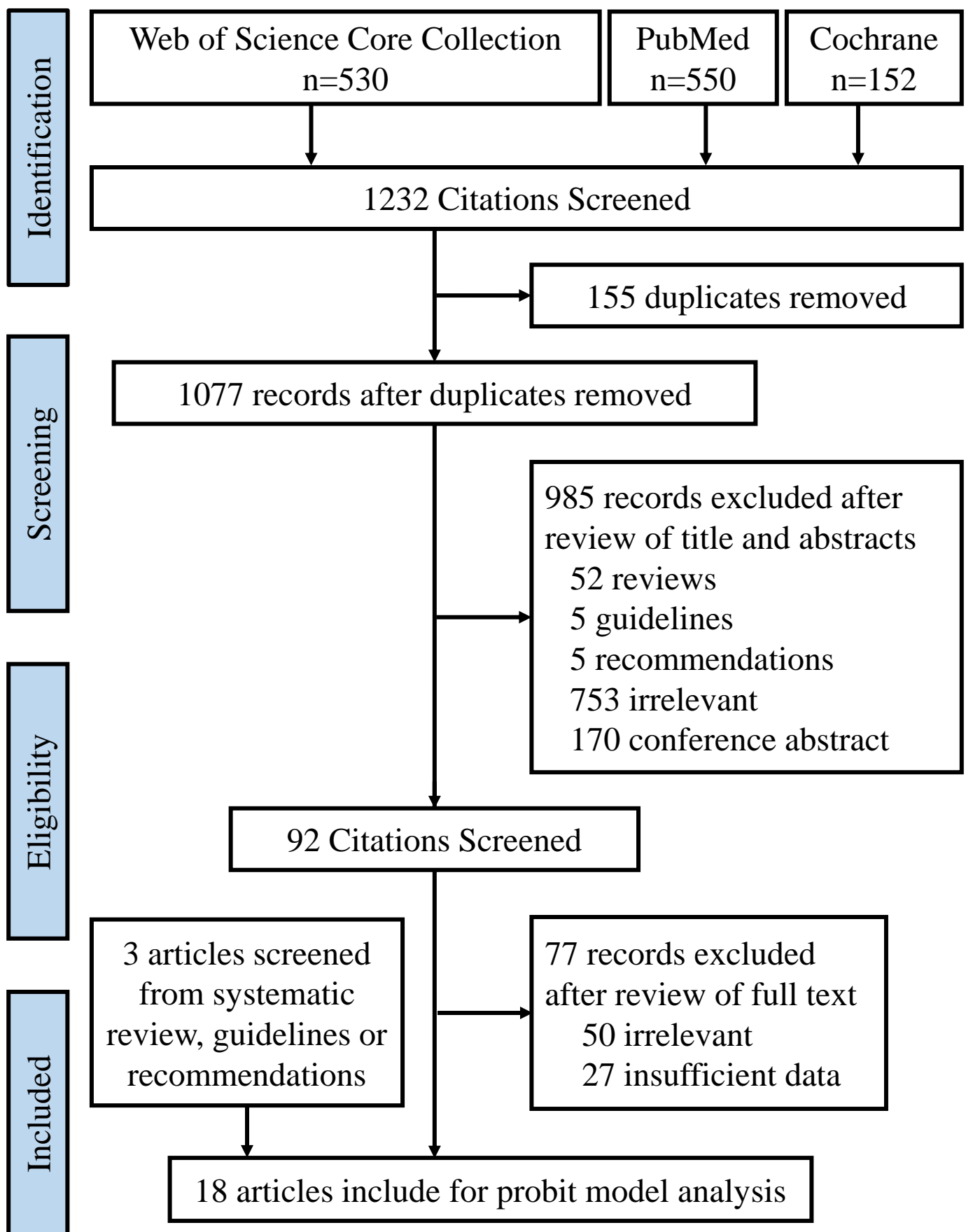

Figure S1. PRISMA Flow diagram of the included studies.

Supplement: Supplementary file 1 — Supplementary Material 1 [file 12885_2024_12486_MOESM1_ESM.pdf]
