# Supplementary material for: Dose-response relationship between volume base dose and tumor local control in definitive radiotherapy for vaginal cancer
Source: BMC Cancer. 2024 Jun 8;24:707. doi: 10.1186/s12885-024-12486-1 (PMC11162573; doi:10.1186/s12885-024-12486-1)
Supplement: Supplementary file 2 — Supplementary Material 2 [file 12885_2024_12486_MOESM2_ESM.pdf]

Table S1. Search strategies of databases.

Databases	Search strategies	Search outcomes
Pubmed	#1 Search Vaginal Neoplasms [MeSH Terms]	5,485
	#2 Search ((((((((((((((((Neoplasm, Vaginal[Title/Abstract]) OR Vaginal Neoplasm[Title/Abstract]) OR Vagina Neoplasms[Title/Abstract]) OR Neoplasm, Vagina[Title/Abstract]) OR Neoplasms, Vagina[Title/Abstract]) OR Vagina Neoplasm[Title/Abstract]) OR Neoplasms, Vaginal[Title/Abstract]) OR Vaginal Cancer[Title/Abstract]) OR Cancer, Vaginal[Title/Abstract]) OR Cancers, Vaginal[Title/Abstract]) OR Vaginal Cancers[Title/Abstract]) OR Cancer of the Vagina[Title/Abstract]) OR Cancer of Vagina[Title/Abstract]) OR Vagina Cancers[Title/Abstract]) OR Vagina Cancer[Title/Abstract]) OR Cancer, Vagina[Title/Abstract]) OR Cancers, Vagina [Title/Abstract])	6,985
	#3 Search (#1) OR #2	9,720
	#4 Search brachytherapy[MeSH Terms]	21,863
	#5 Search ((((((((((((((((((((Radioisotope Brachytherapy[Title/Abstract]) OR Curietherapy[Title/Abstract]) OR Brachytherapy, Radioisotope[Title/Abstract]) OR Plaque Therapy, Radioisotope[Title/Abstract]) OR Radioisotope Plaque Therapy[Title/Abstract]) OR Therapy, Radioisotope Plaque[Title/Abstract]) OR Surface Radiotherapy[Title/Abstract]) OR Radiotherapy, Surface[Title/Abstract]) OR Radiotherapy, Intracavity[Title/Abstract]) OR Intracavity Radiotherapy[Title/Abstract]) OR Radiotherapy, Interstitial[Title/Abstract]) OR Interstitial Radiotherapy[Title/Abstract]) OR Radiotherapy, Implant[Title/Abstract]) OR Implant Radiotherapy[Title/Abstract]) OR High Dose Rate[Title/Abstract]) OR High-dose-rate[Title/Abstract]) OR Low Dose Rate[Title/Abstract]) OR Low-dose-rate[Title/Abstract]) OR Pulsed Dose Rate[Title/Abstract]) OR Pulsed-dose-rate[Title/Abstract]) OR medium-dose-rate[Title/Abstract]) OR medium dose rate[Title/Abstract]	9,182
	#6 Search (#4) OR #5	25,774
	#7 Search (#3) AND #6	654
	Filter: Language: English	550

Web of Science	#1 TOPIC: (Vaginal Neoplasms)	1,257
	#2 TI=(Neoplasm, Vaginal) OR TI=(Vaginal Neoplasm) OR TI=(Vagina Neoplasms) OR TI=(Neoplasm, Vagina) OR TI=(Neoplasms, Vagina) OR TI=(Vagina Neoplasm) OR TI=(Neoplasms, Vaginal) OR TI=(Vaginal Cancer) OR TI=(Cancer, Vaginal) OR TI=(Cancers, Vaginal) OR TI=(Vaginal Cancers) OR TI=(Cancer of the Vagina) OR TI=(Cancer of Vagina) OR TI=(Vagina Cancers) OR TI=(Vagina Cancer) OR TI=(Cancer, Vagina) OR TI=(Cancers, Vagina)	1,510
	#3 #1 OR #2	2,689
	#4 TOPIC: (Brachytherapy)	31,386
	#5 TI=(Radioisotope Brachytherapy) OR TI=(Curietherapy) OR TI=(Brachytherapy, Radioisotope) OR TI=(Plaque Therapy, Radioisotope) OR TI=(Radioisotope Plaque Therapy) OR TI=(Therapy, Radioisotope Plaque) OR TI=(Surface Radiotherapy) OR TI=(Radiotherapy, Surface) OR TI=(Radiotherapy, Intracavity) OR TI=(Intracavity Radiotherapy) OR TI=(Radiotherapy, Interstitial) OR TI=(Interstitial Radiotherapy) OR TI=(Radiotherapy, Implant) OR TI=(Implant Radiotherapy) OR TI=(High Dose Rate) OR TI=(High-dose-rate) OR TI=(Low Dose Rate) OR TI=(Low-dose-rate) OR TI=(Pulsed Dose Rate) OR TI=(Pulsed-dose-rate) OR TI=(Medium-dose-rate) OR TI=(Medium Dose Rate)	7,278
	#6 #4 OR #5	34,319
	#7 #3 AND #6	547
	Filter: Language: English	530
The Cochrane Library	#1 MeSH descriptor: [Vaginal Neoplasms] explode all trees	68
	#2 Neoplasm, Vaginal	295
	#3 Vaginal Neoplasm	295
	#4 Vagina Neoplasms	397
	#5 Neoplasm, Vagina	165

#6 Neoplasms, Vagina	397
#7 Vagina Neoplasm	165
#8 Neoplasms, Vaginal	1,253
#9 Vaginal Cancer	2,206
#10 Cancer, Vaginal	2,206
#11 Cancers, Vaginal	295
#12 Vaginal Cancers	295
#13 Cancer of the Vagina	859
#14 Cancer of Vagina	891
#15 Vagina Cancers	147
#16 Vagina Cancer	891
#17 Cancer, Vagina	891
#18 Cancers, Vagina	147
#19 #1 OR #2 OR #3 OR #4 OR #5 OR #6 OR #7 OR #8 OR #9 OR #10 OR #11 OR #12 OR #13 OR #14 OR #15 OR #16 OR #17 OR #18	2,754
#20 MeSH descriptor: [Brachytherapy]explode all trees	1,095
#21 Radioisotope Brachytherapy	26
#22 Curietherapy	5
#23 Radioisotope Plaque Therapy	15
#24 Surface Radiotherapy	748
#25 Intracavity Radiotherapy	32
#26 Interstitial Radiotherapy	371
#27 Implant Radiotherapy	378
#28 High-dose-rate	555
#29 Low-dose-rate	205

#30 Pulsed-dose-rate	20
#31 Medium-dose-rate	11
#32 #20 OR #21 OR #22 OR #23 OR #24 OR #25 OR #26 OR #27 OR #28 OR #29 OR #30 OR #31	2,702
#33 #19 AND #32	152
